# Angioembolization of Scrotal Arteriovenous Malformations: A Case Report and Literature Review

**DOI:** 10.1155/2020/8373816

**Published:** 2020-02-08

**Authors:** Ammar Mohammad, Wael Sahyouni, Taisser Almeree, Bayan Alsaid

**Affiliations:** ^1^Department of Vascular Surgery, Al-Assad University Hospital, Damascus, Syria; ^2^Department of Radiology, Al-Assad University Hospital, Damascus, Syria; ^3^Department of General Surgery, Al-Assad University Hospital, Damascus, Syria; ^4^Laboratory of Anatomy, Faculty of Medicine, University of Damascus, Damascus, Syria

## Abstract

Arteriovenous malformations (AVMs) of the scrotum are rare lesions, usually diagnosed incidentally during the evaluation of scrotal masses or infertility. It could be presented with acute bleeding or acute pain. We are presenting a case of painless bilateral infiltrated scrotal mass (more advanced in the left side) developed dramatically over a year, no other symptoms existed. The diagnosis was made using duplex ultrasound (DUS), computed tomography arteriography (CTA), and digital subtraction angiography (DSA). Three sessions of angioembolization were performed and followed by surgical resection of the left side of the scrotum.

## 1. Introduction

Scrotal swelling is a very common condition in medical practice. Differential diagnosis includes benign lesions that affect any component of the scrotum (testicles, soft tissues, and vascular plexus), malignancy, metastases, and infection.

Benign vascular lesions are common in adult males. Varicoceles are the most common lesion, and the rarest is arteriovenous malformations (AVMs). These lesions may present in a broad spectrum of symptoms such as swelling, bleeding, acute pain, sensation of heaviness, and mass-like structure.

AVMs are rare congenital abnormal underdeveloped vascular lesions that grow with the child. They present with a mass-like structure with audible or tangible bruit. The duplex ultrasound study shows high systolic flow, low resistive index, and arterialization of venous waveforms. Magnetic resonance imaging (MRI) helps to define the extent of the AVM and the adjacent tissues. Angiograph shows a rapid transit shunting of contrast agent from feeding arteries to enlarged veins through an underdeveloped vascular plexus [1].

The most common locations of arteriovenous malformations (AVMs) are intracranial, followed by extracranial head and neck, extremity, and truncal and visceral sites. However, congenital scrotal AVM is a rare condition [2].

We are reporting a case of huge scrotal AMV managed by angioembolization. We also reviewed data from other similar reported cases in the medical literature.

## 2. Case Presentation

A 19-year-old male patient presented to our center in September 2017 with progressive diffused swelling in the scrotum. The swelling developed dramatically over the previous year with flashing skin and local warmth. There was no pain, urological symptoms, or bleeding. No medical or trauma histories were reported.

**Figure 1:**
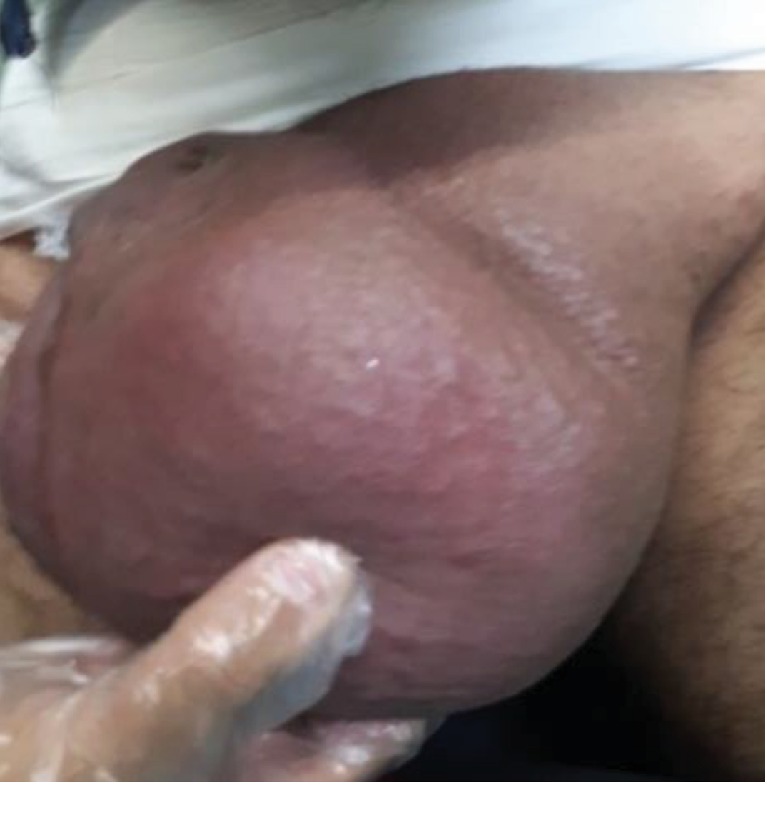
Left scrotal swelling.

Physical examination revealed an asymmetric diffused scrotal swelling (more progressed in the left side) (Figure 1), local warmth, and scrotal skin flashing. By palpation, there was no pulse or thrill. Only the right testicle was palpable.

**Figure 2:**
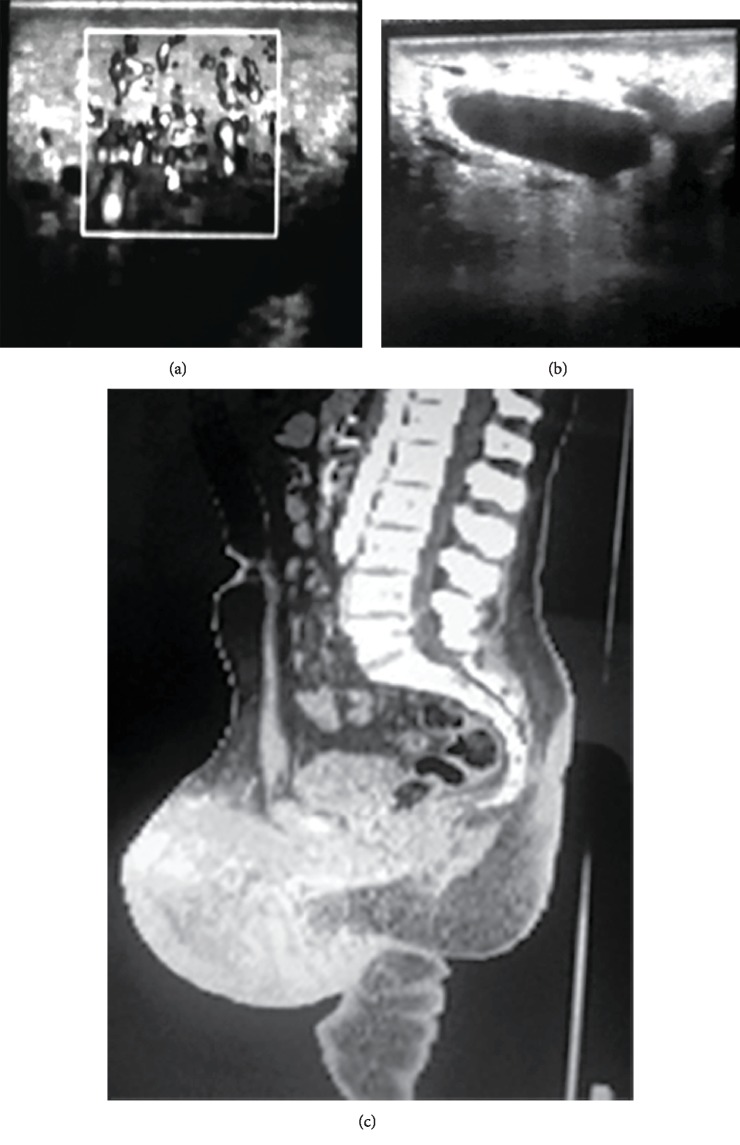
(a, b) Doppler ultrasound showed dilated vessels with both venous and arterial components; (c) computed tomography arteriography.

Duplex ultrasound (DUS) showed prominent vessels exhibiting both venous and arterial components (Figure 2), with unusual high flow velocities, and dilated veins up to 1 cm in width. The skin thickness was 2 mm at its max.

Computed tomography arteriography showed a large AVM in the scrotum occupying almost all the scrotal sac indenting the testicles that look slightly small, the left testicle was encircled by the malformation. The veins seen were also dilated reaching diameters of 1 cm denoting varicoceles.

The AVM has its arterial supply from the internal iliac arteries and at least one of its veins drained in the left common femoral vein.

Digital subtraction angiography (DSA) demonstrated large AVM in the scrotum being centered mainly on the left side with multiple feeding vessels originating from the anterior division of internal iliac and common femoral arteries bilaterally more apparent from the left sides.

The feeding vessels in the left side were the internal pudendal artery, the superficial external pudendal artery arising from the common femoral artery, and the deep external pudendal artery arising from the deep femoral artery.

In the right side, the internal pudendal artery and a small branch of superficial pudendal artery were noticed.

**Figure 3:**
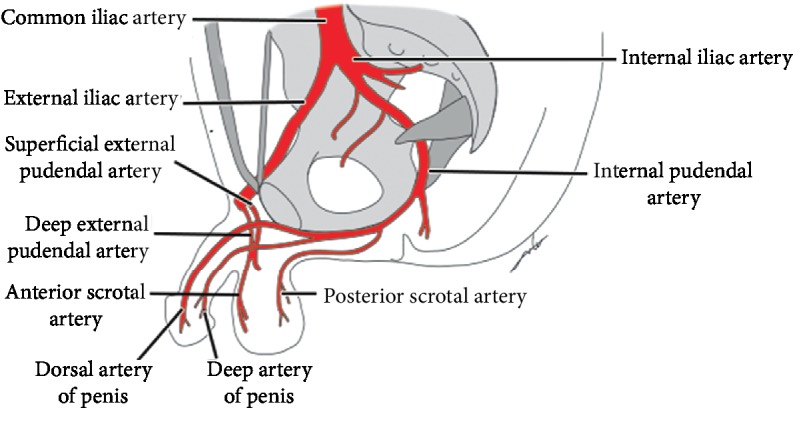
Normal blood supply of scrotum.

The conventional scrotal vascular territory is divided into two parts: anterior one-third and posterior two-thirds. The anterior one-third is supplied by the superficial and deep external pudendal arteries, branching from the femoral artery. The posterior two-thirds is supplied by the internal pudendal artery, branching from the internal iliac artery [3] (Figure 3). The deep external pudendal could be a branch of the deep femoral artery.

Multiple sessions of angioembolization were performed using coils and particles followed by a total resection of the left mass. The patient was informed with all the predictive complications of the procedure.

**Figure 4:**
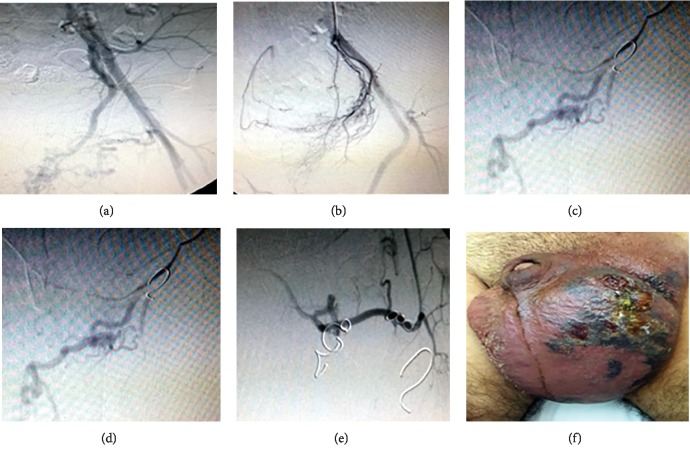
Embolization of multiple vessels: (a, b) internal pudendal artery and (c, d, e) superficial external pudendal artery. (f) View of scrotum after 24 hours.

The first session of embolization was performed in 28/9/2017 in which the left internal pudendal artery and some branches of the superficial external pudendal artery were embolized (Figure 4).

**Figure 5:**
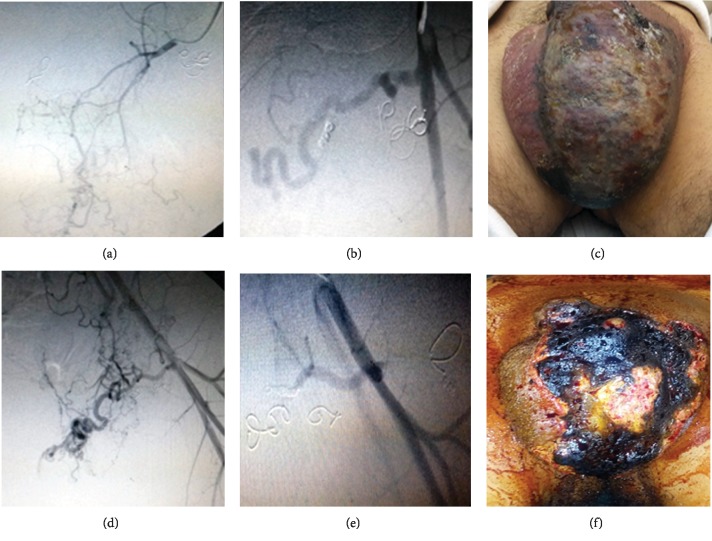
(a, b) Second session of angioembolization of the rest of superficial external pudendal artery branches; (c) affected area after 24 hours; (d) anterioposterior view of left deep external pudendal artery during the third session. (e) Final view showing no more visible feeding branches to the arteriovenous malformation. (f) Final clinically infarcted area before surgery.

In the second session, one week later, the rest of left superficial external pudendal artery branches were embolized (Figures 5(a)–5(c)). A third session of embolization was needed after two weeks to complete the embolization of the left deep external pudendal artery (Figures 5(d)–5(f)).

**Figure 6:**
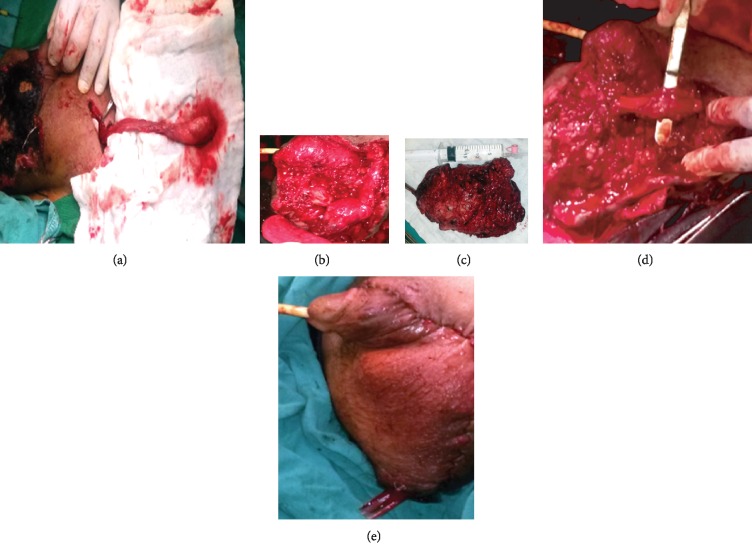
Surgical steps: (a) isolation of left spermatic cord with the testicle from inguinal incision, (b, c) resection of the lesion, and (d) insertion of the left testicle in the right side of scrotum. (e) View after surgery.

Surgical resection was performed in the following day to resect the whole left side of the scrotum, the left testicle was fixed to the right side (Figure 6).

The histopathological study of resected lesions showed vascular vessels with variation in diameters, some hyaline degeneration in the vessels wall and abnormality in arteriovenous structure.

**Figure 7:**
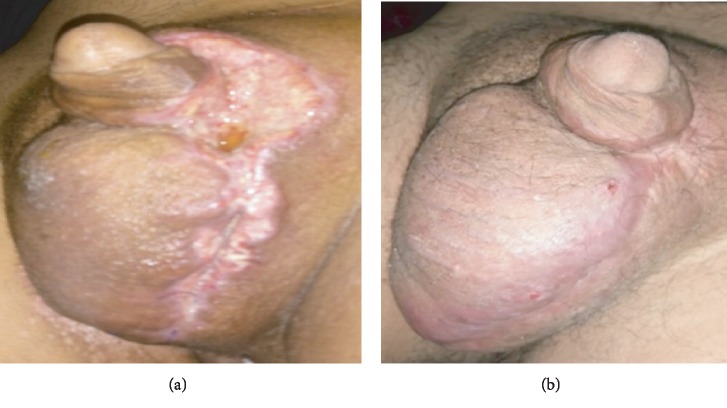
After 3 months (a). After 12 months (b).

The follow-up during the next 12 months was favorable with good healing of the scrotal incision and no recurrence of lesion (Figure 7).

## 3. Literature Review

There are many types of vascular lesions that affect the scrotum or the testicle, Sule et al. [4] divided these into four main types:

1. Varicoceles: common lesions that affect the spermatic cord. They consist of dilated veins with no bruit
2. Hemangiomas: rare lesions that affect the scrotum, consist of dilated veins and capillaries with no bruit detected
3. Lymphangiomas: rare lesions that affect the scrotum, consist of lymphatic ducts with no flow or bruit detected
4. Arteriovenous malformations: very rare lesions that affect the scrotum, consist of abnormal microfistulas (nidus) between arteries and veins with no capillary bed, high flow, and bruit

Scrotal arteriovenous malformations are very rare. Therefore, there have been no clear recommendations on their treatment.

Until this case, there have been 17 published case reports about scrotal AVMs with variation in clinical presentation and medical management which we summarize in Table 1.

## 4. Discussion

Scrotal AVMs are uncommon lesions in medical practice with many types of treatment approaches. There is no medical study to support one approach over another due to the scarcity of this condition. Although embolization of feeding vessels helps in reducing the risk of massive bleeding during surgical resection in most cases, it also raises the risk of radiation complications including malignancy, sperm DNA mutations, and permanent damage to the testicular tissue.

The most common presentation was swelling or infiltrating mass [3–17], followed by pain [4, 5, 11, 13, 16, 17], and bleeding or ulceration [3–5, 8, 11, 14]. Infertility was the primary presentation in few cases [7, 10].

The main diagnostic method was DUS [3–7, 10–14, 16, 17], as we noticed before, followed by DSA [3–5, 7, 10, 12, 14, 16], especially in patients whom treated with endovascular embolization. MRI was used in some cases to confirm the diagnosis [13]. CTA is a helpful method to diagnose the lesion but not effective for establishing the management [15]; therefore, DSA is still the golden standard.

Laboratory tests were limited to the patient's condition. Although sperm analysis was commonly abnormal, it was not a routine test, especially in cases of acute pain or bleeding or with patients who refused to do the test (as in our case).

Percutaneous sclerotherapy into the nidus has been described in low-flow vascular malformation treatment. To our best knowledge, the case written by So et al. is the only case report that described an endovascular method to treat a scrotal AVM with transcatheter coil embolization and percutaneous sclerotherapy. In treating the scrotal lesion with direct puncture, which is a very painful procedure, general anesthesia is required to reduce pain and minimize patient movement. In addition, compression during injection helps in localizing the sclerotherapy and reducing the embolic risk [3].

Embolization materials were variable (coils, gelatin sponge, polyvinyl alcohol sponge, butyl cyanoacrylate mixed with lipodol, particles, and onyx); in our case, we used coils and particles.

Choosing the embolization agent depends on the indication and the experience at hand. Each agent has its specific advantages and disadvantages. Factors influencing the choice of material comprise the size of the target vessel, flow velocity, and duration of embolization (permanent/temporary), see Table 2 [19].

Skin necrosis was described in some cases with angioembolization treatment. Later, surgical approach was needed to remove the necrotic tissue (as in our case).

Some of the possible complications for embolization procedure [20] in pelvic area are as follows:

- Necrosis of the skin, bladder, or other intra-abdominal organs has also been described especially with sever unspecified embolization
- Impotence: bilateral internal pudendal artery embolization
- Arterial perforation
- Hemoglobinuria may develop as a consequence of the hemolysis that follows any sclerosant injection
- Allergic reaction due the use of contrast agent or sclerotherapy agents
- Acute kidney injury due the contrast agent
- Local complications in the insertion artery (hematoma, pseudoaneurysm)
- Delayed malignancy due to the exposure to high dosage of radiation
- DNA mutations of sperm cells/ovarian cells or infertility due to radiation

Conservative approach with periodic assessment was an option in some cases especially in young asymptomatic patients.

## 5. Conclusion

The scarcity of scrotal AVMs and the variety of medical approaches present a challenge in both diagnostic and treatment approaches.

We should think of AVMs in patients with rapidly developed lesions despite other symptoms such as pain or bleeding, without neglecting other deferential diagnoses, mainly tumors and infections.

Doppler US is very helpful for diagnosis of these lesion but is not sufficient for planning the management. Patients should be fully informed about the complications of each treatment plan in order to give an informed consent.

The management should consider the individuality of each case.

## 6. Limitations

We could not do a sperm count (because the patient refused). There was no intervention in the right side of the scrotum due to the hesitation of the patient.

## Figures and Tables

**Table 1:** Published cases till 1/1/2019.

Author (year)	Age (years)	Presentation	Thrill/ bruit	Sperm analysis	Investigation	Management	Follow-up
Bezirdjian et al. (1989) [5]	24	Painless enlarging right scrotal mass	+	Not done	US. Arteriogram	Angioembolization (polyvinyl alcohol sponge (Ivalon)). Surgical debridement	Not mentioned
Hamid et al. (1992) [6]	55	Right scrotal swelling, pain with ulceration and bleeding	+	Azoospermia	Low s.testosterone, DUS, DSA	Angioembolization then surgery	Not commented
Sule et al. (1993) [4]	17	Intermittently bleeding pulsatile left scrotal mass	+	—	DSA	Angioembolization (gelatin sponge and coils) failed. Complete surgical resection was done	No recurrence at 2 years of follow-up
Konus et al. (1999) [7]	8	Progressively enlarging, intermittently bleeding, painful pulsatile scrotal mass	+	Not done	DUS, DSA	Angioembolization (polyvinyl alcohol sponge). Surgical excision	1 year later. No residual disease on follow-up Doppler
Kang et al. (2004) [8]	20	Acute scrotal swelling detected 4 days after a trauma	—	Not done	DUS	Surgical excision. Biopsy showed AVM	Not mentioned
Gonzalez et al. (2002) [9]	31	Left scrotal swelling with virtual azoospermia.	Not mentioned	Azoospermia	DUS, DSA	Bilateral varicocelectomy. Super selective angioembolization followed by surgical excision	3 months of follow-up, sperm analyses improved
Bandi et al. (2004) [10]	67	Recurrent scrotal AVM-bleeding nonhealing ulcer 12 years after preoperative embolization and hemiscrotectomy	Not mentioned	Not done	Not done at second presentation	Surgical excision.	Not mentioned
Choi et al. (2005) [11]		The article was inaccessible					
Monoski et al. (2006) [12]	31	Primary infertility and left scrotal fullness	—	Severe oligospermia	DSA hypertrophied internal pudendal and branch of superficial femoral a.	Bilateral varicocelectomy. Angioembolization. Surgical excision	Sperm count improved. 3 years later, successful spontaneous pregnancy
Yilmaz et al. (2009) [13]	51	Pain and throbbing sensation in right hemiscrotum	Pulsatile vessels +	Not done	Scrotal ultrasound. Confirmed at DUS	Not mentioned	Not mentioned
Jaganathan et al. (2011) [14]	2 32	2 cases both presented with scrotal swelling and bleeding	Not mentioned	Not done	DUS, DSA Emergency DUS, DSA	Selective angioembolization (poly vinyl alcohol). Parents refused surgery. Angioembolization (n-butyl cyanoacrylate mixed with lipodol)	13 months of follow-up, asymptomatic. 18 months of follow-up, no recurrence
Zachariah et al. (2012) [15]	30	Progressive swelling. One episode of acute pain before 4 mo.	—	Not done	DUS, MRI	Angioembolization was rejected. Surgical excision.	Not mentioned
Key R. et al. (2013) [16]	41	Massive bleeding after a hip fracture due to trauma. With large right retro peritoneal hematoma	—	—	DUS, DSA	Several sessions of angioembolization (micro coils/emposphere's/gel foam particles/onyx	18 weeks later, no symptoms
Sato et al. (2013) [17]	38	Recurrent scrotal mass	—	—	CTA, biopsy (micro-AV fistula—AVM)	Surgical excision	10 months later, no recurrence
So WL et al. (2014) [3]	26	Scrotal pain and swelling	—	Not mentioned	DUS (AVM). DSA	Coil embolization. Subcutaneous sclerosant (sodium tetradecyl sulfate 3% with ethiodised oil (2 : 1 ratio)	3 months of review, no recurrence
Muslim et al. (2014) [18]	16	Right scrotal swelling associated with mild pain	Not mentioned	Not mentioned	DUS	Refused embolization. Surgical excision with dissecting the spermatic cord through an inguinal incision to protect it	One-year follow-up, no recurrence
Our case	19	Left scrotum swelling	+	Not performed	DUS, MCT, DSA	Three sessions of embolization, resection of scrotal lesion	One-year follow-up, no recurrence

**Table 2:** Types of embolization agents.

Embolization agents	
Organic	Autologous clots, fibrin
Coils	Standard coils, detachable coils, active coils
Plugs, balloons	Detachable balloon, vascular plug
Liquids, sclerosants	Alcohol, polymerizing substances (histoacryl), detergents (e.g., fibrovein, ethoxysclerol), antibiotics (e.g., doxocycline, bleomycin), precipitating substances (onyx®)
Particles	Gelfoam, polyvinyl alcohol particles, spherical particles (e.g., spherical PVA, acryl polymere)
